# Rational Modification of a Cross-Linker for Improved Flexible Protein Structure Modeling

**DOI:** 10.1021/acs.analchem.4c05319

**Published:** 2025-01-09

**Authors:** Iakovos Saridakis, Kish R. Adoni, Thomas Leischner, Bogdan R. Brutiu, Saad Shaaban, Giammarco Ferrari, Konstantinos Thalassinos, Nuno Maulide

**Affiliations:** † Institute of Organic Chemistry, University of Vienna, Währinger Straße 38, 1090 Wien, Austria; ‡ Institute of Structural and Molecular Biology, Division of Biosciences, University College London, Darwin Building Room 101A, London WC1E 6BT, United Kingdom; ◪ Research Platform NeGeMac, University of Vienna, 1090 Vienna, Austria

## Abstract

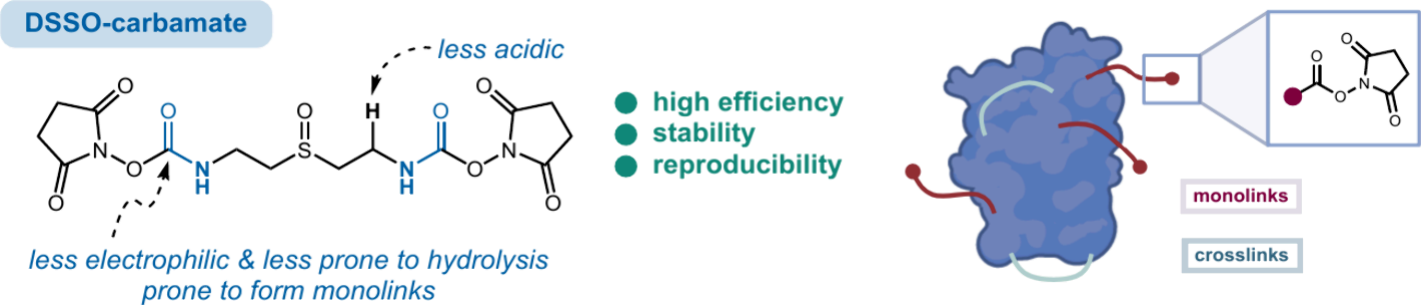

Chemical cross-linking/mass spectrometry (XL-MS) has emerged as a complementary tool for mapping interaction sites within protein networks as well as gaining moderate-resolution native structural insight with minimal interference. XL-MS technology mostly relies on chemoselective reactions (cross-linking) between protein residues and a linker. DSSO represents a versatile cross-linker for protein structure investigation and in-cell XL-MS. However, our assessment of its shelf life and batch purity revealed decomposition of DSSO in anhydrous solution via a retro-Michael reaction, which may reduce the active ingredient down to below 90%. To mitigate the occurrence of this degradative mechanism, we report the rational design and synthesis of DSSO-carbamate, which contains an inserted nitrogen atom in the DSSO backbone structure. This modification to DSSO yielded remarkably favorable stability against such decomposition, which translated to higher cross-link and monolink recovery when performing XL-MS on monomeric flexible proteins. Recently, XL-MS has been leveraged against AlphaFold2 and other protein structure prediction algorithms for improved prediction of flexible monomeric multiconformational proteins. To this end, we demonstrate that our novel cross-linker, termed DSSO-carbamate, generated more accurate protein structure predictions when combined with AlphaFold2, on account of its increased recovery of cross-links and monolinks, compared to DSSO. As such, DSSO-carbamate represents a useful addition to the XL-MS community, particularly for protein structure prediction.

Protein structure determination plays a pivotal role in elucidating biological processes in modern molecular biology. A plethora of techniques are part of the repertoire to this end, including X-ray crystallography, nuclear magnetic resonance spectroscopy, and cryo-electron microscopy.^1^ These methods, however, require large amounts of purified protein. In contrast, cross-linking mass spectrometry (XL-MS)^2−4^ has recently emerged as an alternative and complementary tool for identifying protein–protein interactions (PPI) (Figure 1A) or uncovering protein structure in living cells, requiring comparatively smaller amounts of the protein of interest (Figure 1B).^4−8^ To this end, several novel cross-linking reagents have been reported, made commercially available, and have become commonplace within the XL-MS community during the past decade. Well-established and widely used cross-linkers such as disuccinimidyl sulfoxide (DSSO),^9^ disuccinimidyl dibutyric urea (DSBU),^10^ azide-tagged, acid-cleavable disuccinimidyl bis-sulfoxide (azide-A-DSBSO),^11^ (NNP9),^12^ and (d_0_/d_10_-DMDSSO)^13^ have been designed and tuned for determination of protein structure and the elucidation of protein–protein interactions (Figure 1C). The typical workflow of the XL-MS part marries obtaining mass spectrometry experimental data with computational analysis using high-level algorithms, which tend to be tailored for each linker through considerable optimization.^9−13^ Within the XL-MS community, DSSO (**5**) is among the most commonly used cross-linkers, as reported by Scheltema and co-workers.^14^

**Figure 1:**
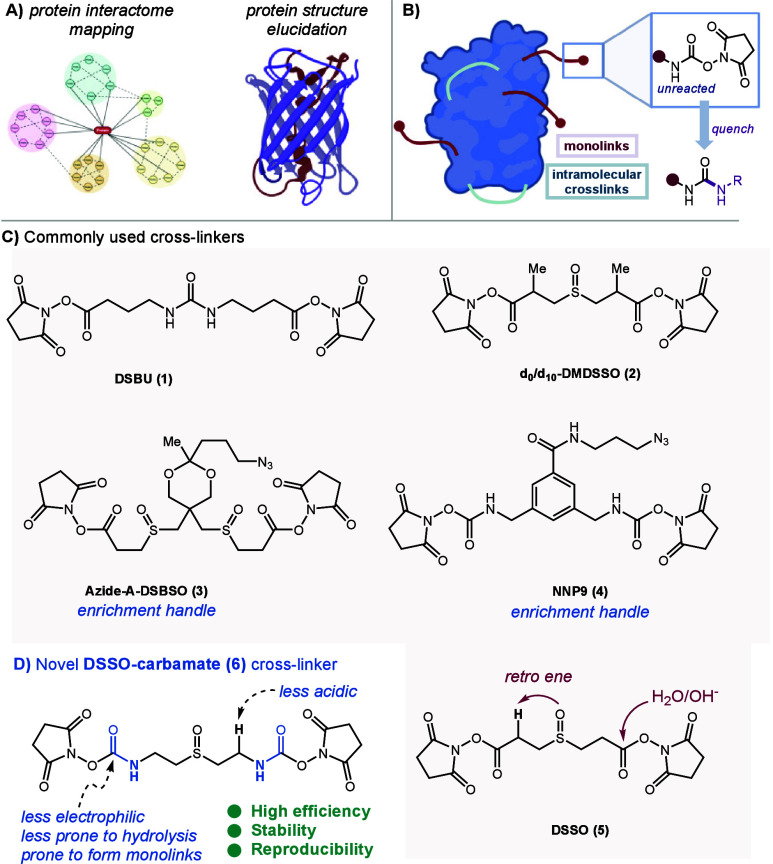
(A) Illustrative impact of XL-MS in proteomics and structure elucidation, (B) schematic representation of monolinks and cross-links, (C) commonly used cross-linkers, and (D) the design of a novel carbamate linker.

Despite the success of chemical cross-linkers in protein biochemistry analysis, degradation of DSSO in anhydrous solution (DMSO), as identified from our NMR stability assays, can reduce the active ingredient down to below 90% of the original starting material. Our work demonstrates that insertion of a nitrogen atom into the molecular structure of DSSO, termed DSSO-carbamate, leads to enhanced chemical stability of the cross-linker against degradation in anhydrous solution. Consequently, our analysis revealed improved cross-link and monolink recovery when analyzing monomeric flexible proteins using our DSSO-carbamate cross-linker, compared to standard DSSO. As such, we found that our DSSO-carbamate complemented the application of XL-MS integrated AlphaFold2 protein structure prediction algorithms, facilitating the prediction of more accurate protein structures, when compared to standard DSSO.

## Experimental Section

### LC-MS Sample Preparation

Glutamine binding protein (QBP) was acquired and purified as described in Manalastas-Cantos et al.^19^ Human 20S Proteasome complex (h20S) was purchased from Biotechne, catalogue number: E-360. All samples were buffer exchanged into 20 mM 4-(2-hydroxyethyl)-1-piperazineethanesulfonic acid (HEPES) buffer, pH 7.5 at 4 °C using an Amicon Ultra-0.5 Centrifugal 10 kDa filter unit (purchased from Merk), followed by dilution to 5.00 μM using 20 mM HEPES. For glutamine cofactor bound QBP, 5 mM glutamine was added to the sample prior to cross-linking. Disuccinimidyl sulfoxide (DSSO) and disuccinimidyl sulfoxide-carbamate (DSSO-carbamate) were dissolved in dimethyl sulfoxide (DMSO) to create a stock concentration of 50 mM, before 10 μL of each cross-linker was added to the protein sample to generate a 1:100 protein:cross-linker ratio. Samples were incubated for 60 min before quenching with 50 mM NH_4_HCO_3_. Samples were then reduced at 37 °C for 45 min using 8 mM dithiothreitol (DTT) before alkylation in darkness for 30 min with 20 mM iodoacetamide. Samples were then trypsin digested (1:50 trypsin to protein ratio, *w*/*w*) with incubation overnight at 37 °C. Digestion was quenched to a final trifluoroacetic acid concentration of 0.50% (*v*/*v*) before samples were Ziptip desalted (purchased from Merk) and reconstituted in 0.10% formic acid to a final peptide concentration of 1 mg/mL.

### LC-MS Setup: Human 20S Proteasome Complex

Three experimental replicates were analyzed for DSSO and DSSO-carbamate. An UltiMate 3000 RSLCnano liquid chromatography system (Thermo Fisher Scientific), with a 50 cm μPAC Neo HPLC analytical column (COL-NANO050NEOB) and a 0.075 × 20 mm trap cartridge (Acclaim PepMap C18 100 Å, 3 μm), was connected to a SilicaTip emitter. Column temperature was set at 45 °C, and column flow rate was set to 300 nL/min. Mobile phase A (0.1% formic acid, 3.2% acetonitrile) and mobile phase B (96.8% acetonitrile/0.1% formic acid) were applied with an elution gradient from 5.0 to 35.0% mobile phase B over 35 min. Total run time per sample was 60 min. High-field asymmetric waveform ion mobility spectrometry (FAIMS) was applied between the nano-ESI and MS with internal stepping across −50.0, −60.0, and −70.0 V over 3 s, and the FAIMS carrier gas flow rate was set to 4.6 L/min. A Thermo Fisher Scientific Orbitrap Eclipse Tribrid mass spectrometer was used for this work. The mass spectrometer was externally calibrated using a Pierce FlexMix calibration solution. nanoESI was performed by the application of a voltage to a SilicaTip emitter via an HPLC liquid junction cross. Spray stability and intensity was optimized by varying the SilicaTip electrospray voltage (1–2 kV) and varying SilicaTip positioning (in the *x*, *y*, and *z* dimensions). Transfer capillary temperature was set to 275 °C, RF lens was set to 40%, precursor ion mass spectrum was acquired at a resolution of 120,000 with a mass range of 380 to 1400 *m*/*z* and a precursor ion charge state range of 3+ to 8+. MS1 spectra were recorded with a data-dependent analysis Top20 method; automatic gain control was set to a target of 400,000 (100%); maximum injection time mode was set to Auto; precursor ions were isolated with a 1.6 *m*/*z* window using the quadrupole mass filter; monoisotopic precursor selection was enabled. For Orbitrap mass analysis based MS2 acquisition, higher-energy collision-induced dissociation was applied with a normalized energy of 30%. Orbitrap resolution was set to 30,000; scan range was set to define first mass (120 *m*/*z*); automatic gain control was set to 200%; maximum injection time mode was set to dynamic.

### LC-MS Setup: QBP

Two experimental replicates, each consisting of two technical replicates, were run for DSSO and DSSO-carbamate. An UltiMate 3000 RSLCnano liquid chromatography system (Thermo Fisher Scientific), with a 50 cm μPAC Neo HPLC analytical column (COL-NANO050NEOB) and a 0.075 mm × 20 mm trap cartridge (Acclaim PepMap C18 100 Å, 3 μm), was connected to a SilicaTip emitter. Column temperature was set at 45 °C, and column flow rate was set to 750 nL/min. Mobile phase A (0.1% formic acid, 3.2% acetonitrile) and mobile phase B (96.8% acetonitrile/0.1% formic acid) were applied with an elution gradient from 5.0 to 35.0% mobile phase B over 20 min. Total run time per sample was 30 min. A Thermo Fisher Scientific Orbitrap Eclipse Tribrid mass spectrometer was used for this work. The mass spectrometer was externally calibrated using PierceTM FlexMixTM calibration solution. nanoESI was performed by the application of a voltage to a SilicaTip emitter, via an HPLC liquid junction cross. Spray stability and intensity were optimized by varying the SilicaTip electrospray voltage (1–2 kV) and varying SilicaTip positioning (in the *x*, *y*, and *z* dimensions). Transfer capillary temperature was set to 275 °C; RF lens was set to 40%; precursor ion mass spectrum was acquired at a resolution of 60,000 with a mass range of 350 to 1600 *m*/*z* and a precursor ion charge state range of 2+ to 6+. MS1 spectra were recorded with a data-dependent analysis Top20 method; automatic gain control was set to a target of 400,000 (100%); maximum injection time mode was set to Auto; precursor ions were isolated with a 1.4 *m*/*z* window using the quadrupole mass filter; monoisotopic precursor selection was enabled. For Orbitrap mass analysis-based MS2 acquisition, higher-energy collision-induced dissociation was applied with a normalized energy of 30%. Orbitrap resolution was set to 15,000; scan range was set to define first mass (120 *m*/*z*); automatic gain control was set to 200%; maximum injection time mode was set to auto.

### Data Analysis and Processing

All RAW files were processed with Proteome Discoverer 2.5 from Thermo Fisher Scientific using the Sequest HT and XlinkX nodes. Default settings were selected, unless otherwise specified. For analysis of h20S proteasome, the *Homo sapiens* 20S Proteasome FASTA file was downloaded from Uniprot. Sequest HT was selected with modifications including dynamic methionine oxidation (+15.995 Da), static cysteine carbamidomethylation (+57.021 Da), dynamic N-terminal acetylation (+42.0106 Da), dynamic methionine loss, and N-terminal acetylation (−89.030 Da). For analysis of DSSO monolinks: lysine DSSO amidated (+175.030 Da) was included, and for the analysis DSSO-carbamate monolinks: lysine DSSO-carbamate amidated (+205.052 Da) were selected. Percolator node was used with a false discovery rate (FDR): 0.01 (target-decoy method). For cross-link analysis, XlinkX node was selected with Target-Decoy PSM Validator and Xlink/PD Detect parameters set to Acquisition strategy MS2, Cross-link modification DSSO (+158.004 Da, K) and DSSO-carbamate (+188.026 Da, K), XlinkX PD Search parameters set to *Homo sapiens* FASTA file (July 2022), XlinkX PD Validator parameters set to FDR threshold 0.01, and separate inter from intra: true.

For analysis of QBP, a FASTA file containing protein accession: P0AEQ3, and 200 randomly selected protein sequences from *Homo sapiens* Uniprot version July 2022, was used. Sequest HT was selected with modifications, including dynamic methionine oxidation (+15.995 Da), static cysteine carbamidomethylation (+57.021 Da), dynamic N-terminal acetylation (+42.0106 Da), dynamic methionine loss, and N-terminal acetylation (−89.030 Da). For analysis of DSSO monolinks: lysine DSSO amidated (+175.030 Da) was included and for the analysis DSSO-carbamate monolinks: lysine DSSO-carbamate amidated (+205.052 Da) were selected. Fixed Value PSM Validator node was used with false discovery rate (FDR): 0.01 (target-decoy method). For cross-link analysis, XlinkX node was selected with Target-Decoy PSM Validator and Xlink/PD Detect parameters set to Acquisition strategy nonCleavable_fast, Cross-link modification DSSO (+158.004 Da, K) and DSSO-carbamate (+188.026 Da, K), XlinkX PD Search parameters set to P0AEQ3, and 200 randomly selected protein sequences from *Homo sapiens* Uniprot FASTA file (July 2022), XlinkX PD Validator parameters set to FDR threshold 0.01 and separate inter from intra: true. All identified cross-links and monolinks were then selected for further analysis.

XL-MS Alphafold2 protein structure prediction was performed on QBP using all identified cross-links and monolinks from DSSO and DSSO-carbamate, with workflow performed as described in Manalastas-Cantos et al.^19^ Highest scoring predicted structures were aligned to the QBP open and cofactor bound crystal structures, PDB: 1GGG and 1WDN, respectively. Structure alignment was performed using the matchmaker tool in ChimeraX, to generate an RMSD value as a measure of accuracy of the structure prediction (version 1.7.1).^26^

All peptide and protein identification information has been deposited on ProteomeXchange, Proteomics Identifications Database (PRIDE) data set identifier: PXD051742; username: reviewer_pxd051742@ebi.ac.uk; password: x4btLr1f).

### General Procedures

All reactions were performed in round-bottom flasks or vials fitted with rubber septa and magnetic stirring unless otherwise stated. Reaction vessels were flushed with argon prior to use unless otherwise stated. Liquids and solutions were transferred via a syringe. All reactions were performed using anhydrous solvents obtained from Acros Organics, TCI, or Sigma-Aldrich. Reaction progress was monitored by thin layer chromatography (TLC) performed on aluminum plates coated with silica gel F_254_ with 0.2 mm thickness. Chromatograms were visualized by fluorescence quenching with UV light at 254 nm or by staining with potassium permanganate, followed by heating. Flash column chromatography was performed using silica gel 60 (230–400 mesh, Merck and Co.) or prepacked columns and reagent grade solvents.

### Materials

All commercial reagents and solvents were used without further purification.

### Instrumentation

All ^1^H NMR, ^13^C DEPTQ-135 NMR, ^13^C CPD NMR, and ^19^F NMR spectra were recorded using a Bruker AV-400, AV-500, AV-600, or AV-700 spectrometer at 300 K. Chemical shifts (δ) are given in parts per million (ppm), referenced to the solvent peak of CDCl_3_, defined at δ = 7.26 ppm (^1^H NMR) and δ = 77.16 ppm (^13^C NMR) and the solvent peak of DMSO-*d*_6_, defined at δ = 2.50 ppm (^1^H NMR) and δ = 39.52 ppm (^13^C NMR).^27^ Coupling constants (*J*) are reported in Hertz (Hz). ^1^H NMR splitting patterns are designated as singlet (s), doublet (d), triplet (t), quartet (q), quintet (quint), or a combination thereof, as they appeared in the spectrum. If the appearance of a signal differs from the expected splitting pattern, the observed pattern is designated as apparent (app). Splitting patterns that could not be interpreted or easily visualized are designated as multiplet (m) or broad (br). Infrared (IR) spectra were obtained using PerkinElmer Spectrum 100 FT-IR spectrometer. Wavenumbers (ν_max_) are reported in cm^–1^. Mass spectra were obtained using a Bruker maXis UHR-TOF spectrometer (70 eV), using electrospray ionization (ESI) or atmospheric-pressure chemical ionization (APCI) or an Agilent 7200B GC/Q-TOF spectrometer (70 eV), and using electron ionization (EI). Optical rotations were measured on a PerkinElmer 341 polarimeter using a 100 mm path-length cell at 589 nm (*c* given in g/(100 mL)). Details of the chromatographic conditions are indicated under each compound.

### Synthesis of DSSO-carbamate Precursor: *Bis*-(2,5-dioxopyrrolidin-1-yl) (thio-*bis*(ethane-2,1-diyl))dicarbamate

In a flame-dried 50 mL round-bottom flask equipped with a magnetic stirring bar, commercially available *N*,*N*′-disuccinimidylcarbonate (553 mg, 2.05 mmol, 2.05 equiv) was suspended in 10 mL of dry CH_3_CN under Ar atmosphere. Next, commercially available 2,2′-thio-*bis*-(2-ethylamine) (123.0 mg, 1.00 mmol, 1.00 equiv) was added dropwise, resulting in the formation of a clear colorless solution within a few minutes. The reaction mixture was stirred at 23 °C for 16 h. Subsequently, all volatiles were removed under reduced pressure, and the crude product in the form of a sticky residue was dissolved in 250 mL of DCM. The organic phase was quickly washed with distilled water (3 × 50 mL), and all volatiles were removed under reduced pressure after drying over Na_2_SO_4_, yielding the title compound in 60% yield (120 mg).

### Synthesis of DSSO-carbamate: *Bis*-(2,5-dioxopyrrolidin-1-yl) (sulfinylbis(ethane-2,1-diyl))dicarbamate

In a flame-dried 10 mL round-bottom flask equipped with a magnetic stirring bar, **8** (50.0 mg, 0.12 mmol, 1.00 equiv) was dissolved in 2 mL of dry CH_3_CN under an Ar atmosphere. To the obtained colorless solution was slowly added 30% aq. H_2_O_2_ (12.4 μL; 0.12 mmol; 1.00 equiv) dropwise, and the reaction mixture was stirred for 1 h at 22 °C. All volatiles were removed under reduced pressure subsequently, yielding the title compound in sufficient purity as a white, crystalline solid in 98% yield (51 mg).

Further details of syntheses, including reaction schemes and NMR data, are provided in the Supporting Information.

## Results and Discussion

In general, state-of-the-art linkers (Figure 1C) can be assessed based on four key factors: stability, efficiency, biorthogonality (when an enrichment handle is present), and synthetic accessibility. Developing a diverse array of linkers that can address these factors and fill existing gaps in the chemical space remains an important objective.

While most of the literature in XL-MS focuses on cross-links between protein residues at an appropriate distance, more recently the supporting role of so-called monolinks has started to receive attention.^15^ Though experimental cross-linked sites have been leveraged to improve protein structure predictions,^15^ it has been reported that between 8% and 25% of theoretically possible cross-links within a monomeric protein are recovered from XL-MS experiments.^15−17^ Conversely, monolink recovery has been described to reach between 50% and 80% from all theoretically possible monolinks, suggesting their enhanced compatibility with integrative protein structure determination algorithms.^15^ Despite this, monolinks are typically discarded as undesired background peptides when processing and analyzing XL-MS data.^18^ Recently, Topf, Thalassinos, and co-workers demonstrated that the incorporation of experimental cross-links and monolinks into their AlphaFold2 protein structure determination algorithm, with the application of a shallow multiple sequence alignment, can improve the accuracy of protein structure prediction via more effective ranking of predicted structures within the ensemble. Further, they demonstrated the necessity of monolink data when applying the algorithm to small, multiconformational globular proteins such as glutamine binding protein (QBP), whose structure is modified by cofactor binding.^19^

The popularity of DSSO within the XL-MS community^14^ is attributed to its small size (minimized perturbance and decreased loop-links) as well as to its symmetrical dialkysulfoxide backbone which enables facile collision-induced dissociation (CID) upon MS-MS acquisition for protein structure determination. While its applications are wide-ranging and well-documented,^20−25^ to the best of our knowledge there are no detailed reports concerning its shelf stability or batch purity. This fact is surprising given the well-known hydrolytic sensitivity of NHS esters. During ongoing work, we became acutely aware of the chemical lability of DSSO when analyzing either commercial samples or those prepared in-house. As detailed below, much of this lability can be chemically connected to the electrophilicity of the NHS moiety (**5**, Figure 1C).

### Insertion of a Nitrogen Atom into DSSO Improved Cross-Linker Stability

We hypothesized that a net nitrogen atom “insertion” between the reactive sites and the backbone could at once render the resulting linker less prone to decomposition, while its potentially reduced electrophilicity might allow for a higher incidence of monolinks. In this work, we present a chemical stability study on DSSO as a showcase, leading to the rational design of a novel reagent, which we term “DSSO-carbamate” (**6**) (Figure 1D). We show that **6** offers dramatically enhanced integrity in solution, resulting in higher efficiency in cross-link and monolink generation.

**Figure 2:**
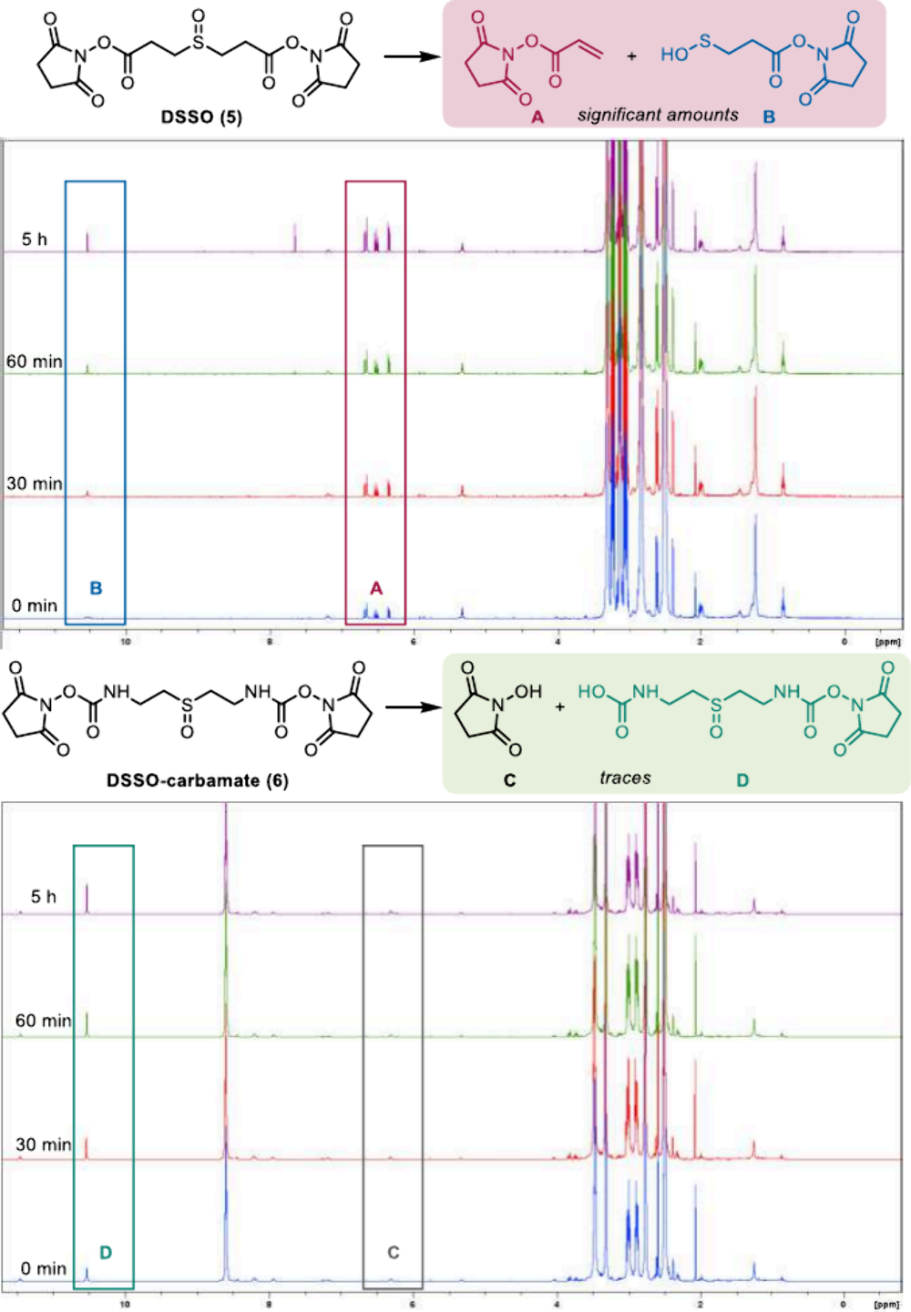
Chemical stability studies of DSSO and DSSO-carbamate in DMSO.

During preliminary investigations (Figure 2, see the SI for more details), we could determine that the main mechanism for decomposition of DSSO in anhydrous solution is a retro-Michael reaction^28^ (alternatively describable as retro-ene sulfoxide elimination), leading to formation of NHS-acrylate **A** and sulfenic acid **B** (Figure 2, top). Indeed, upon standing in DMSO solution, DSSO decomposes up to 12% into (**A**+**B**) within 5 h. It is important to note that this decomposition might not impede the reactivity of (preserved) DSSO with protein nucleophilic residues and, thus, does not significantly interfere with XL-MS determination. Nevertheless, it is remarkable that slightly aged samples of a commercially available, widely used chemical reagent may indeed contain less than 90% of the actual active ingredient. More importantly, the retro-Michael products can lead to false-positives upon MS2 data analysis.^9^

Conversely, in DSSO-carbamate (**6**) the aforementioned decomposition is effectively shut down (Figure 2, bottom), leading to a high chemical integrity of the reagent in solution. Indeed, an additional effect of nitrogen insertion is that delocalization of electrons from said nitrogen onto the carbonyl’s antibonding orbital renders the resulting functional group considerably less electrophilic.

**Scheme 1:**
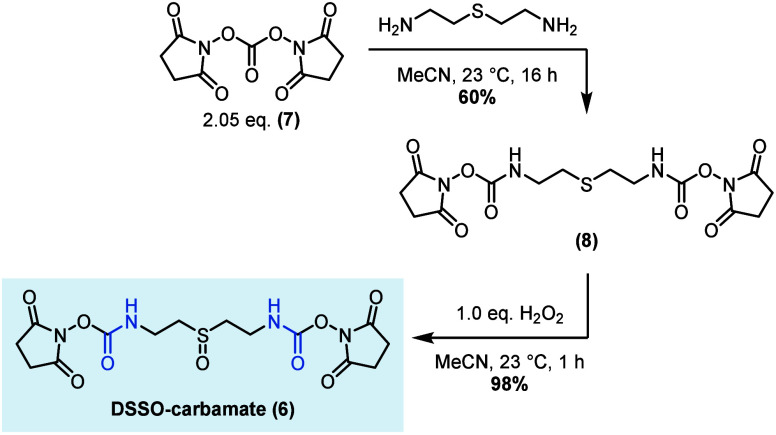
Synthesis of DSSO-carbamate

From a synthesis perspective, DSSO-carbamate (**6**) is readily accessed in 2 steps from di(*N*-succinimidyl)-carbonate (**7**, Scheme 1). Given its much-increased chemical stability in solution, we hypothesized that the increased relative amount of active ingredient may result in the recovery of more cross-links and monolinks when performing XL-MS experiments.

### DSSO-carbamate Improved Monolink Recovery from h20S Proteasome Complex

**Figure 3:**
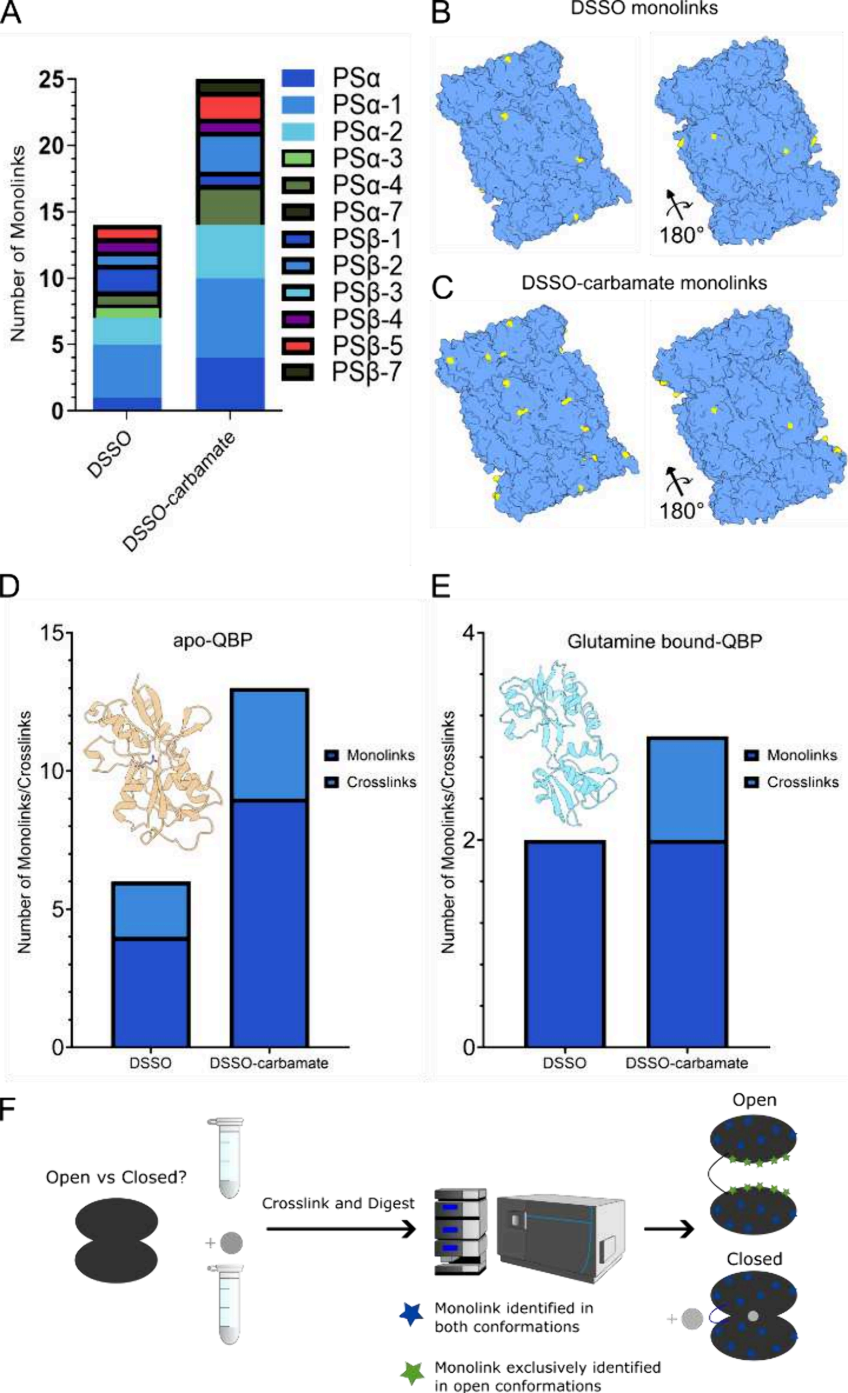
DSSO vs DSSO carbamate for XL-MS-based protein structure determination. Identification of lysine-monolinks from XL-MS analysis of human 20S Proteasome complex. Bar chart annotated with proteasome subunits (PS) (A). Mapping of identified lysine-monolinks (yellow) from DSSO and DSSO carbamate on human 20S Proteasome complex (PDB: 6RGQ) (B, C). Identification of lysine-monolinks and cross-links from DSSO and DSSO-carbamate XL-MS analysis of Glutamine Binding Protein monomer in apo-conformation (PDB: 1GGG) (D) and glutamine-bound conformation (PDB: 1WDN) (E). Workflow for the application of XL-MS for the investigation of protein conformational changes upon cofactor binding (F).

Monolinks that are identified from XL-MS experiments provide valuable auxiliary information pertaining to the associated residue’s depth and solvent accessibility within a protein. Thus, for a protein that can occupy multiple conformations, differential monolink identifications can shed light on how the protein’s conformation changes with different external conditions such as cofactor binding (Figure 3F).^15,19^ Considering the reduced decomposition of DSSO-carbamate, with preferential reaction via NHS ester hydrolysis toward protein-lysine ε-amine groups, we proposed that these properties may positively impact monolink recovery during XL-MS experiments for protein structural analysis, while also generating the required cross-links to deconvolute conformational changes of flexible proteins.

To initially assess monolink recovery within an XL-MS workflow, DSSO and DSSO-carbamate were compared for the XL-MS analysis of the human 20S Proteasome complex (h20S), which comprises 28 subunits of 14 different protein subunit isoforms, without any cross-link or monolink enrichment performed in the sample preparation workflow. Twenty-five monolinked lysine residues were identified across 9 h20S subunits from DSSO-carbamate, compared to 14 residues across 9 h20S subunits recovered from DSSO. These identified monolinks were then mapped onto the h20S crystal structure (PDB: 6RGQ)^17^ to demonstrate the superior monolink-derived structural information that was extracted from DSSO-carbamate based XL-MS analysis (Figure 3B, C). Monolink data from DSSO and DSSO-carbamate for h20S Proteasome are provided in the Supplementary Table. Interestingly, more cross-links were identified with DSSO vs DSSO-carbamate, perhaps indicating DSSO as the preferential interprotein cross-linker of supramolecular protein complexes. All cross-link identifications also reported in the Supplementary Table.

### DSSO-carbamate Improved Cross-Link and Monolink Recovery from Small Flexible Protein QBP

We next investigated the complementarity of DSSO-carbamate for an XL-MS integrated AlphaFold2 workflow, as described by Topf, Thalassinos, and co-workers.^19^ While AlphaFold has revolutionized the field of protein structure prediction, intrinsically disordered regions and flexible proteins that can occupy multiple conformations still pose a challenge to its accuracy when predicting protein structures. To this end, we reasoned that the improved recovery of monolinks, without impeding cross-link recovery, from DSSO-carbamate, compared to DSSO, would provide more structural information for such multiconformational proteins, and this data could then be leveraged against XL-MS integrated AlphaFold2 pipelines.^19^

XL-MS was performed on glutamine binding protein (QBP), a 27.2 kDa globular protein that compacts upon binding its glutamine cofactor.^28^ 9 monolinks were recovered from apo-QBP with DSSO-carbamate, compared to 4 monolinks with DSSO. Upon QBP compaction with glutamine cofactor, 2 monolinks were identified from both DSSO-carbamate and DSSO. The recovery of more monolinks from the apo-condition can be reasoned by the extended structure of QBP, exposing more solvent-accessible lysine residues for interaction with the cross-linker. Interestingly, despite no cross-link enrichment in the sample preparation workflow, the apo-QBP condition identified 4 cross-links with DSSO-carbamate compared to 2 with DSSO. The glutamine bound QBP conformation recovered one cross-link from DSSO-carbamate, and no cross-links were identified from DSSO (Figure 3D,E). The recovery of more cross-links from DSSO-carbamate, as well as more monolinks, suggests its complementarity for the structural investigation of small globular proteins. Together, these data suggested DSSO-carbamate to be a useful addition to the cross-linker toolbox for XL-MS integrated protein structure determination algorithms.

### DSSO-carbamate Generated Improved Performance of XL-MS Integrated AlphaFold2 Protein Structure Prediction

**Figure 4:**
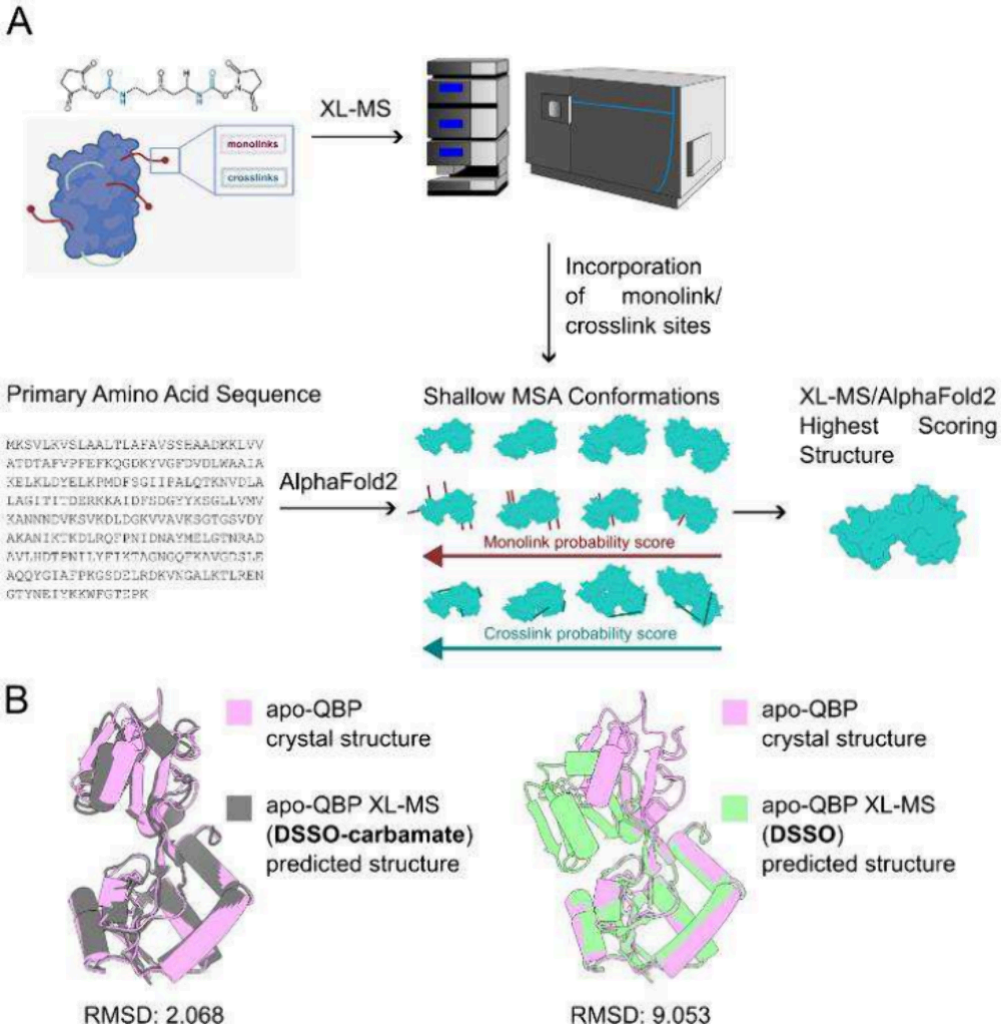
Investigating the complementarity of DSSO-carbamate vs DSSO, for XL-MS integrated AlphaFold2 protein structure prediction. XL-MS was performed on QBP in both its apo-form and when bound to glutamine cofactor, before LC-MS/MS based analysis to extract monolinks and cross-links. These data were then imputed into the XL-MS integrated AlphaFold2 pipeline, as described by Topf, Thalassinos, and co-workers.^19^ All predicted structures from the shallow multiple sequence alignment were scored against experimentally identified monolinks and cross-links before the highest scoring structure was selected from the ensemble (A). For the apo-QBP structure, the highest scoring structure from the ensemble, generated with DSSO-carbamate, was 4.4-fold more accurate than that generated with DSSO, based on RMSD alignment of the predicted structure to the known crystal structure of apo-QBP (B).

We next performed XL-MS integrated Alphafold2 predictions on QBP, using our recovered monolinks and cross-links from DSSO-carbamate and DSSO (Figure 4A). When comparing the highest scoring structure predictions from the two cross-linkers, DSSO-carbamate generated an apo-QBP structure with an RMSD of 2.068 from the apo-QBP crystal structure, compared to 9.053 for DSSO (Figure 4B). Further, while the cofactor-bound QBP predicted structure resulted in an RMSD of 3.190 from the cofactor-bound QBP crystal structure for DSSO-carbamate, DSSO did not provide enough monolink/cross-link events to generate a predicted structure. These data demonstrate the enhanced capacity of DSSO-carbamate to complement XL-MS integrated protein structure determination algorithms, compared to DSSO, driven by the superior recovery of cross-links and monolinks for DSSO-carbamate vs DSSO. It should be noted that the XL-MS integrated Alphafold2 package uses cross-linker length parameters corresponding to BS^3^/DSS, which would impact cross-link identification ranking within the ensemble of predicted conformations. All QBP monolink and cross-link identifications are provided in the Supplementary Table.

## Conclusions

We report herein the development of DSSO-carbamate as a novel cross-linker. Our work demonstrates the enhanced stability of the DSSO-carbamate by the insertion of a nitrogen atom into the cross-linker-backbone, resulting in more relative active ingredient when performing XL-MS. This represents a valuable addition to the field of structural proteomics, specifically enhancing the utility of cross-links and monolinks in protein structure elucidation, particularly for small flexible proteins that can occupy multiple conformations depending on their environment. Indeed, our findings underscore the critical role of monolinks, often overlooked in traditional cross-linking approaches, in providing valuable insights into protein conformation and interaction dynamics. The increased recovery of cross-links and monolinks facilitated by DSSO-carbamate not only supports more accurate protein modeling when integrated with cutting-edge computational algorithms like XL-MS-AlphaFold2 but also opens new avenues for exploring protein dynamics and interactions in a more nuanced manner. We believe this work could pave the way for further innovations in cross-linker chemistry and protein structural analysis.

## Supporting Information Available

Molecular structures and reaction schemes corresponding to DSSO-carbamate precursor and DSSO-carbamate, together with peak identifications from ^1^H NMR, ^13^C NMR, IR (neat), and high-resolution MS; ^1^H NMR spectra for DSSO-carbamate and DSSO-carbamate precursor; ^1^H NMR spectra corresponding to DSSO and DSSO-carbamate degradation in *d*_*6*_-DMSO, every 30 min for 300 min; table containing all identified cross-links and monolinks from QBP and h20S complex (PDF)

## Abbreviations

XLMS: cross-linking mass spectrometry
LC-MS: liquid chromatography–mass spectrometry
h20S: human 20S proteasome complex
QBP: glutamine-binding protein
MSA: multiple sequence alignment
