# Supplementary material for: Rational Modification of a Cross-Linker for Improved Flexible Protein Structure Modeling
Source: Anal Chem. 2025 Jan 9;97(2):1273–80. doi: 10.1021/acs.analchem.4c05319 (PMC11755394; doi:10.1021/acs.analchem.4c05319)
Supplement: Supplementary file 1 — ac4c05319_si_001.pdf [file ac4c05319_si_001.pdf]

Rational Modification of a Cross-Linker for Improved Flexible Protein Structure Modeling

Iakovos Saridakis,[1,†] Kish R. Adoni,[2,†] Thomas Leischner,[1,†] Bogdan R. Brutiu,[1]
Saad Shaaban,[1] Giammarco Ferrari, Konstantinos Thalassinos[2,*] and Nuno Maulide[1,*]

[1] *University of Vienna, Institute of Organic Chemistry, Währinger Straße 38, 1090 Vienna, Austria*

E-mail: nuno.maulide@univie.ac.at

[2] *University College London, Institute of Structural and Molecular Biology, Division of Biosciences, Darwin Building Room 101A, London WC1E 6BT*

E-mail: k.thalassinos@ucl.ac.uk

Table of Contents

Synthesis of DSSO-carbamate precursor 3
Synthesis of DSSO-carbamate 4
NMR Spectra 5
NMR Stability Studies 7
Replicate analysis for 20S Proteasome mononlinks 12
References 12

Synthesis of DSSO-carbamate precursor

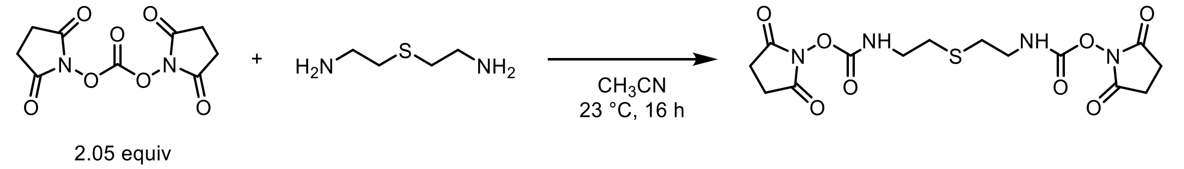

***Bis*-(2,5-dioxopyrrolidin-1-yl) (thio-*bis*(ethane-2,1-diyl))dicarbamate (8)**

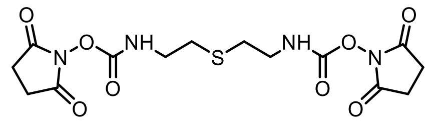

^{1}H NMR (400 MHz, CD_3CN) δ 6.52 (s, 2H), 3.27 (q, J = 6.5 Hz, 4H), 2.65 (s, 8H), 2.60 (t, J = 6.8 Hz, 4H) ppm.

^{13}C NMR (176 MHz, CD_3CN) δ 171.8 (4C), 153.0 (2C), 42.1 (2C), 31.6 (2C), 26.2 (4C) ppm.

IR (neat) ν_{max}: 2957, 2922, 2852, 1805, 1777, 1730, 1706, 1527,1466, 1428, 1388, 1365, 1296, 1258, 1200, 1100, 1070, 1045, 1017, 993, 890, 812, 751, 729, 646, 619, 556, 431 cm^{-1}.

HRMS (ESI^{+}): exact mass calculated for $[M+Na]^{+}$ ($C_{14}H_{18}N_4O_8SNa^{+}$) requires *m/z* 425.0738, found *m/z* 425.0738.

Synthesis of DSSO-carbamate

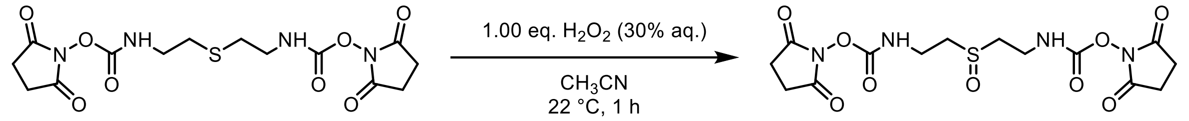

***Bis*-(2,5-dioxopyrrolidin-1-yl) (sulfinylbis(ethane-2,1-diyl))dicarbamate (6)**

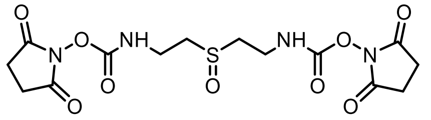

^{1}H NMR (600 MHz, DMSO-*d*$_6$) δ 8.59 (t, J = 5.5 Hz, 2H), 3.47 (dd, J = 12.5, 5.9 Hz, 4H), 3.01 (dt, J = 14.9, 7.6 Hz, 2H), 2.93 – 2.85 (m, 2H), 2.77 (s, 8H) ppm.

^{13}C NMR (151 MHz, DMSO-*d*$_6$) δ 171.8 (4C), 153.0 (2C), 42.1 (2C), 31.6 (2C), 26.2 (4C) ppm.

IR (neat) ν_{max}:.1788, 1777, 1739, 1721, 1529, 1430, 1259, 1236, 1205, 1154, 1112, 1077, 1054, 1008, 897, 818, 765 cm^{-1}.

HRMS (ESI$^+$): exact mass calculated for $[M+Na]^+$ ($C_{14}H_{18}N_4O_9SNa^+$) requires *m/z* 441.0687, found *m/z* 441.0686.

Note 1: Sample contains some free *N*-hydroxysuccinimide (δ = 2.60 ppm) originating from partial hydrolysis of the starting material in ^{1}H NMR spectra. All efforts to remove it failed.

Note 2: Uncorrectable phase shimming for DMSO-solvent signal between 39.7 – 39.9 ppm in ^{13}C NMR spectra.

NMR Spectra

***Bis*-(2,5-dioxopyrrolidin-1-yl) (thio-*bis*(ethane-2,1-diyl))dicarbamate (8)**

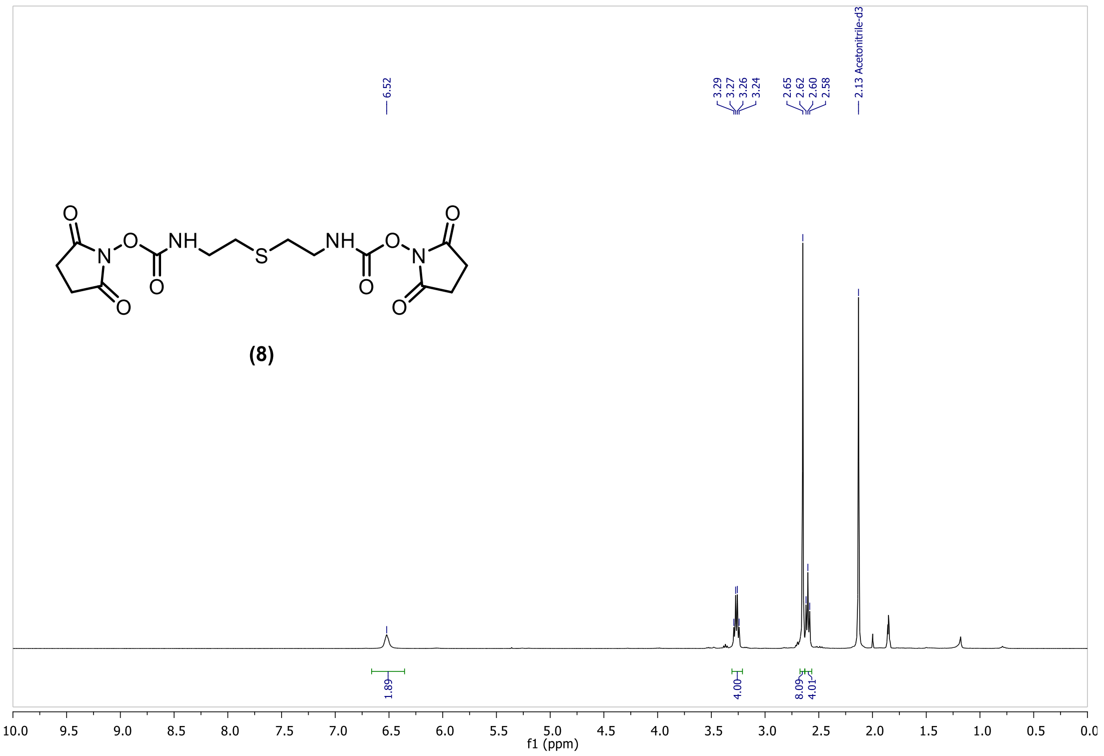

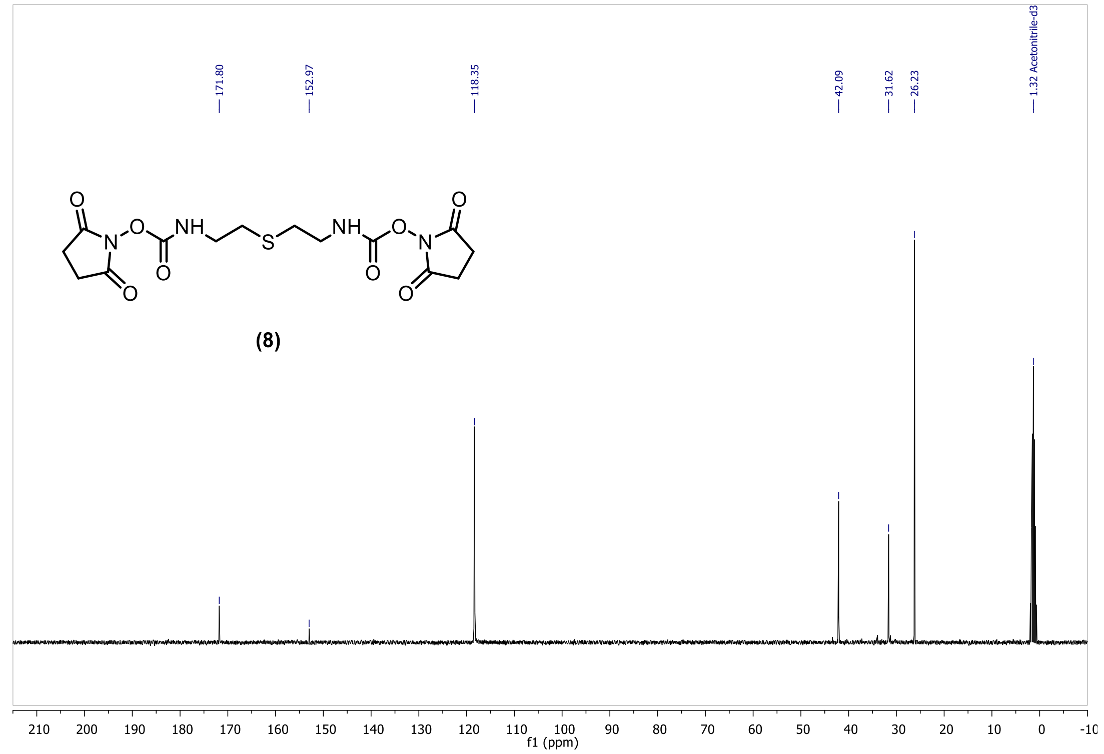

Note: Uncorrectable phase shimming for DMSO-solvent signal between 39.7 – 39.9 ppm.

Bis-(2,5-dioxopyrrolidin-1-yl) (sulfinylbis(ethane-2,1-diyl))dicarbamate (DSSO-carbamate, 6)

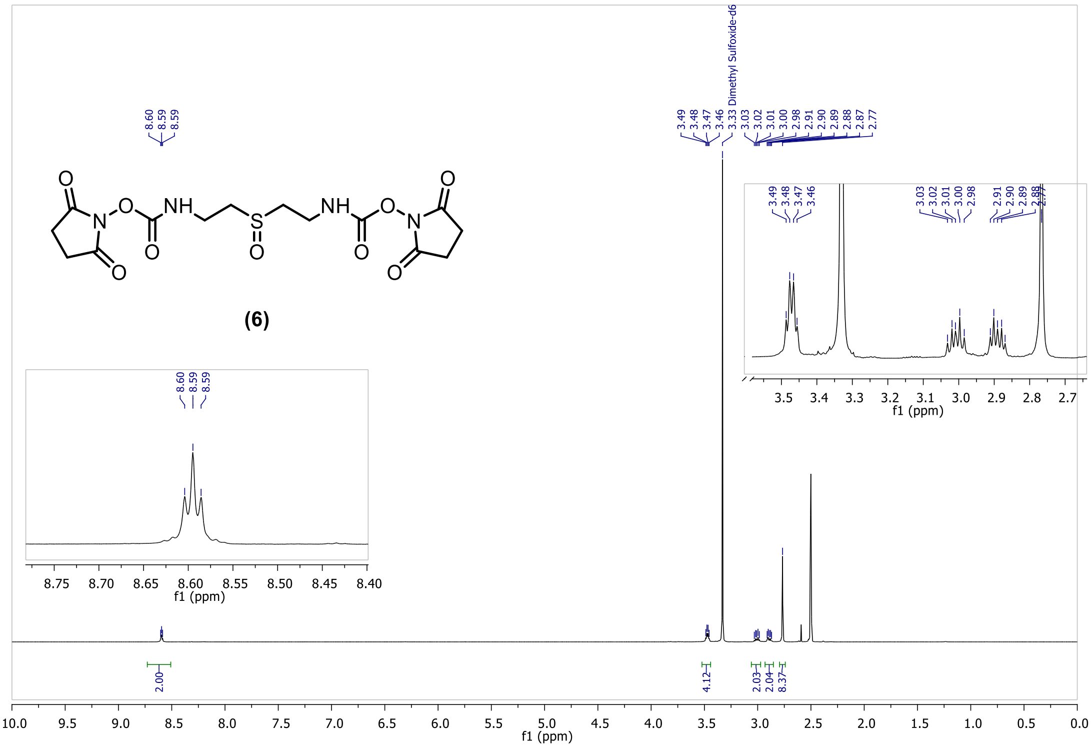

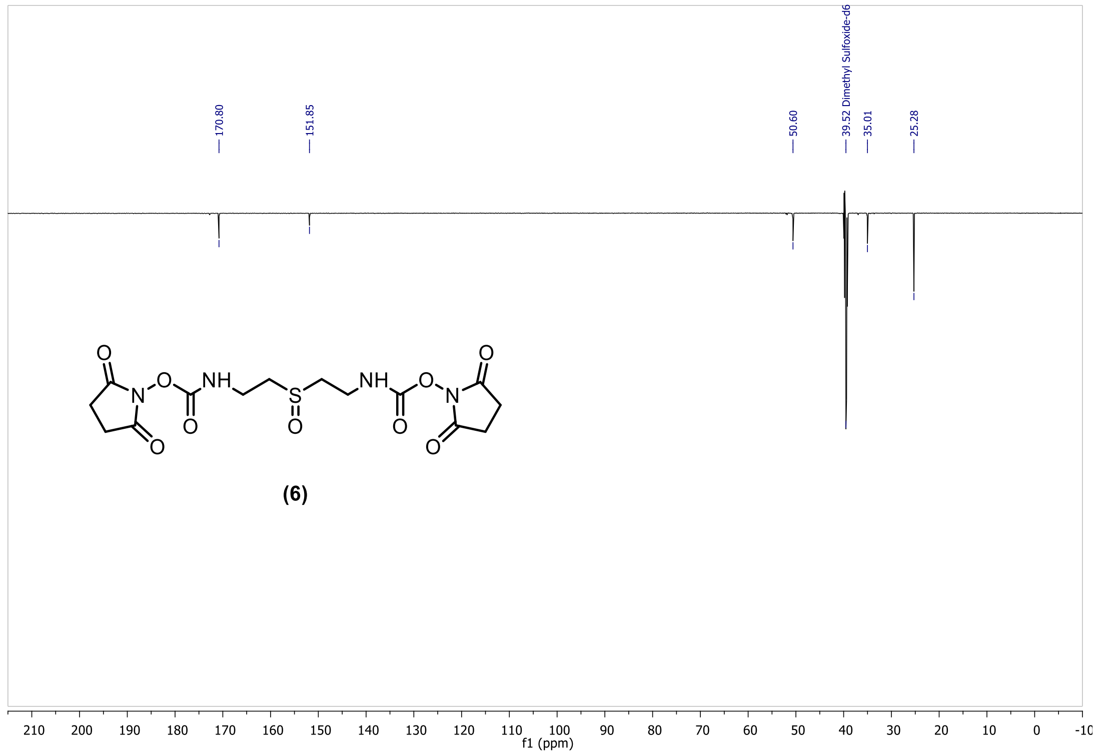

NMR Stability Studies

Stability Study of DSSO

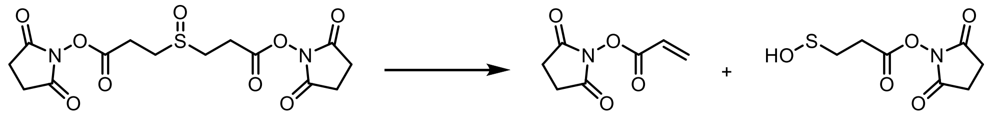

DSSO **A** **B**

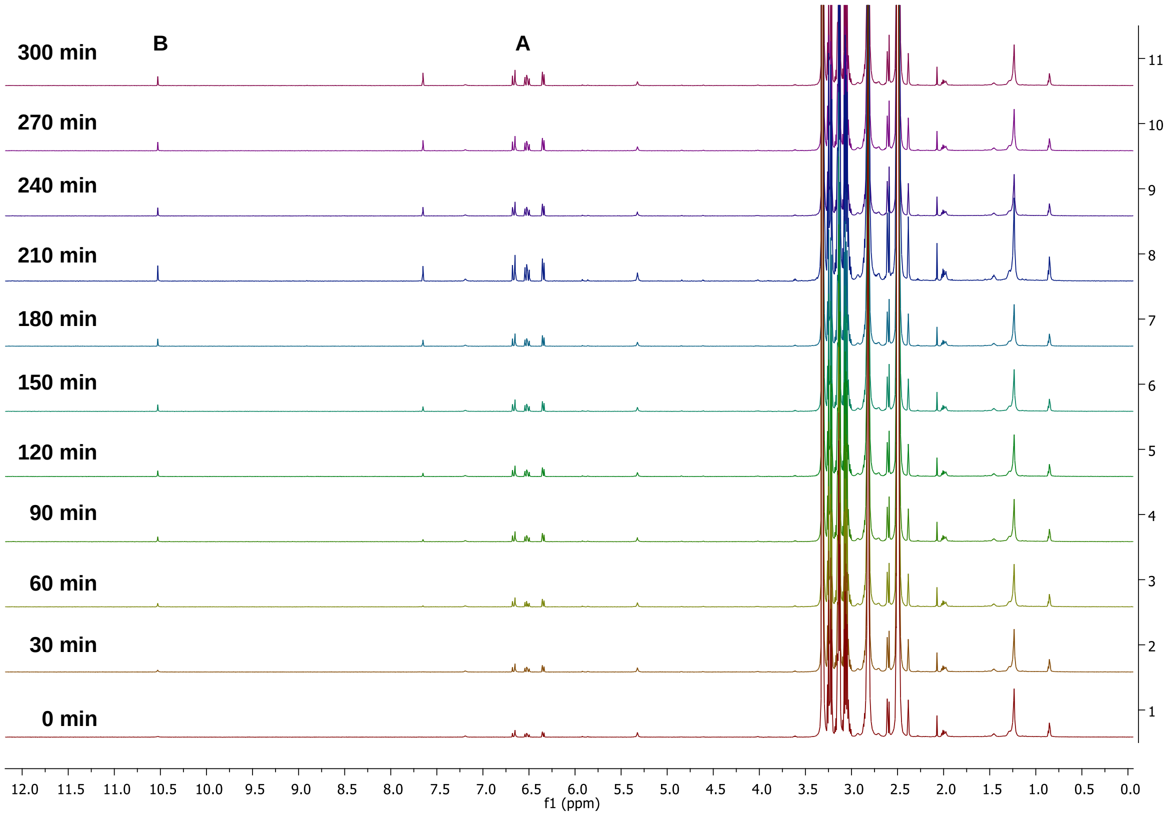

The chemical stability of commercially available DSSO in d_6-DMSO was investigated. For this purpose, one milligram of a commercially available sample (Thermo Fisher) was dissolved in a freshly opened bottle of the NMR solvent. The sample was analyzed by ^{1}H NMR in intervals of 30 minutes over in total 6 h. Interestingly, the degradation of DSSO, forming the Michael acceptor **A** and the sulfenic acid **B**, seems to start right after dissolving the material as indicated by the proton NMR at t = 0 min. This process continues and after 6 h, significant degradation of the starting material was observed. Despite having reactive NHS-ester groups, prone to hydrolysis, this decomposition pathway was not observed in significant amounts and hence seems only to contribute to a minor extend to the degradation of DSSO.

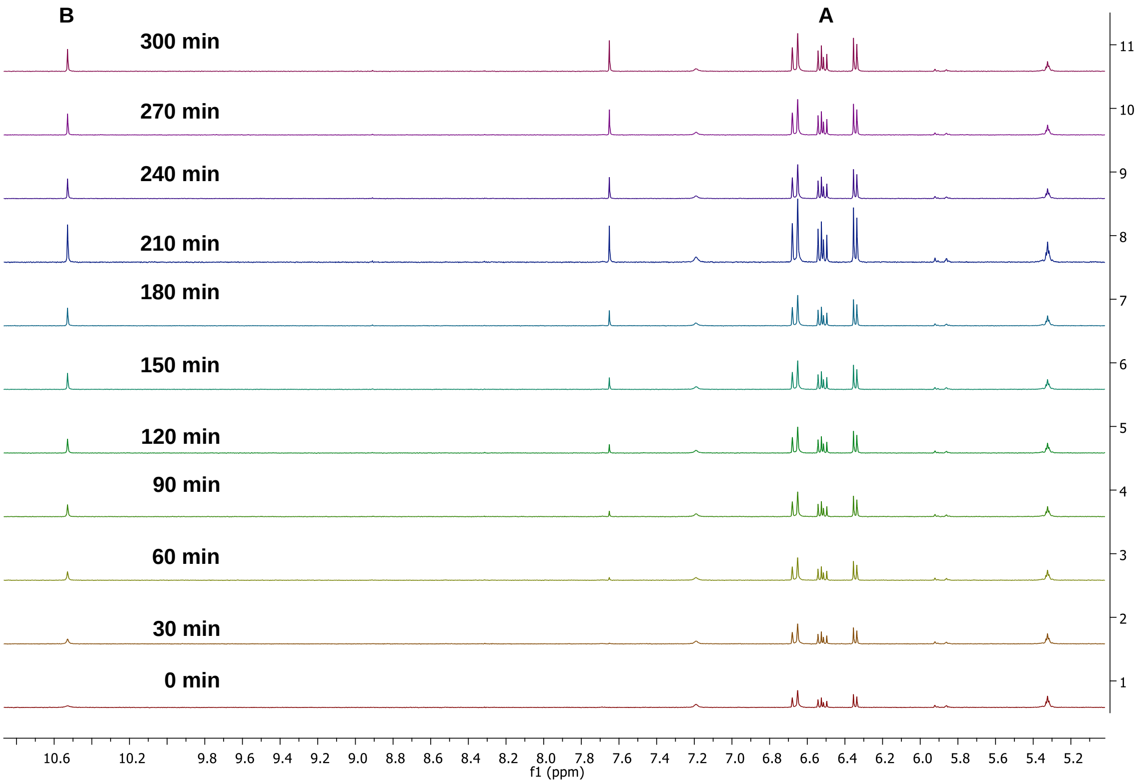
B
A
300 min
270 min
240 min
210 min
180 min
150 min
120 min
90 min
60 min
30 min
0 min
f1 (ppm)

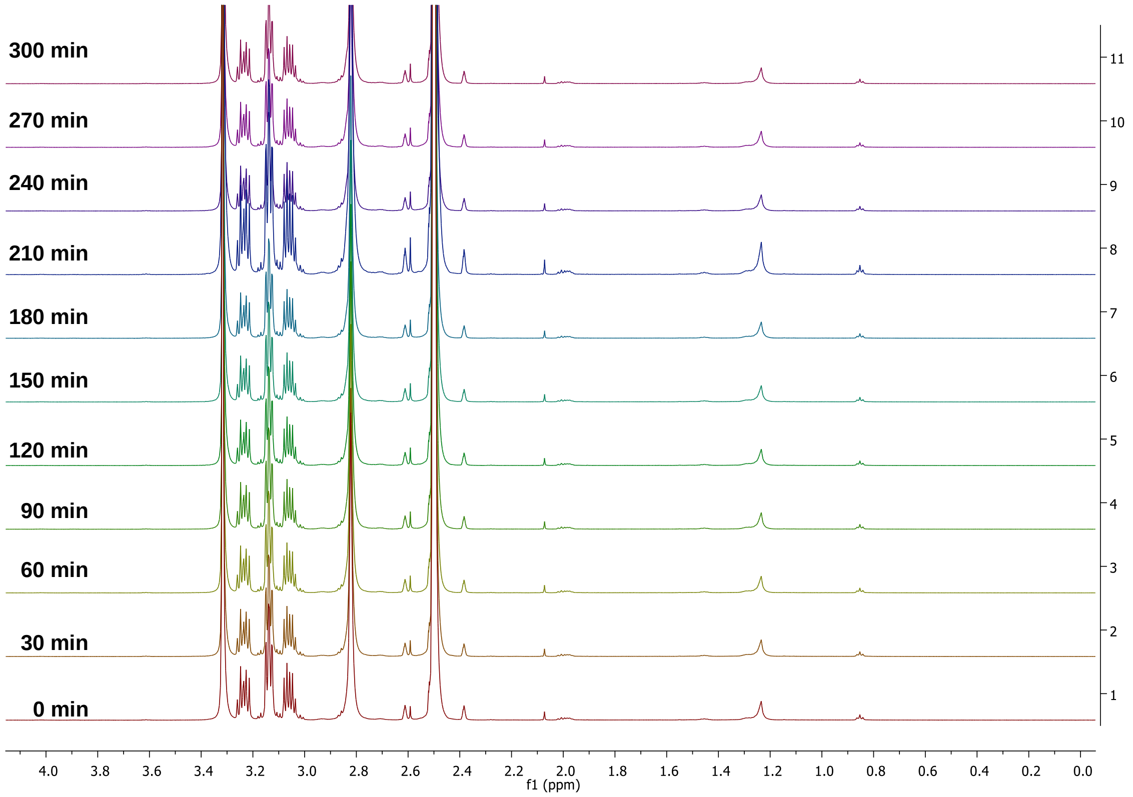
300 min
270 min
240 min
210 min
180 min
150 min
120 min
90 min
60 min
30 min
0 min
f1 (ppm)

Procedure: 1 mg of commercially DSSO (Thermo Fisher Scientific; Provided by the group of Prof. K. Mechtler, IMP, Vienna, Austria) was dissolved in 0.7 mL of fresh DMSO-d_6. Next, ^{1}H NMR spectra were recorded every 30 minutes for overall 6 h.

Stability Study of DSSO-carbamate (6)

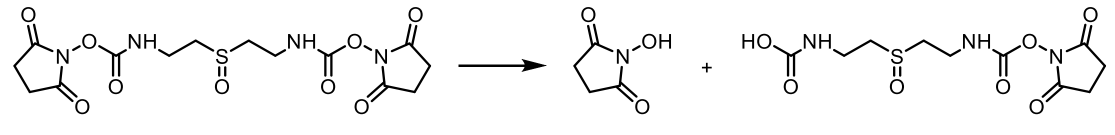

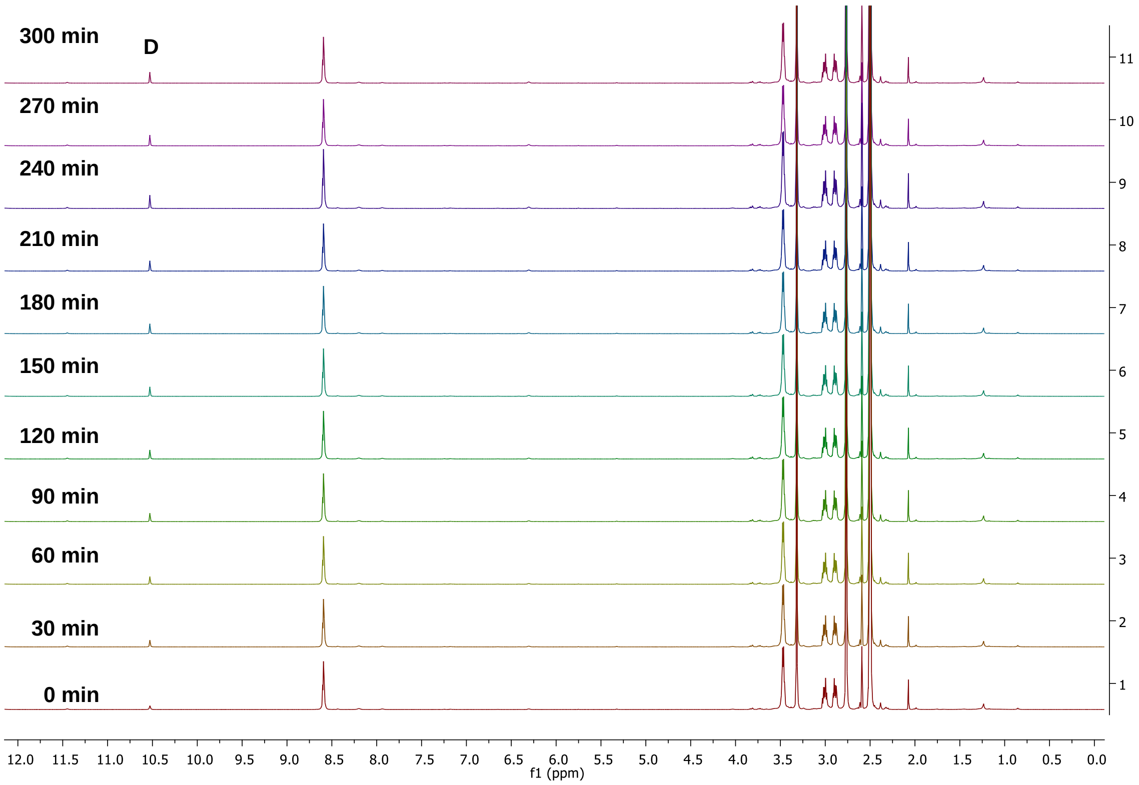

One milligram of DSSO-carbamate (**6**) was dissolved in a freshly opened bottle of DMSO-d_6. The sample stability was monitored by ^{1}H NMR in intervals of 30 minutes over in total 6 h. The introduction of an additional NH moiety, thereby transforming the esters in DSSO to carbamates in DSSO-carbamate, indeed efficiently suppressed the sample decomposition via the formation of a sulfenic acid and a Michael acceptor as observed for DSSO. The only degradation pathway that we were able to observe for DSSO-carbamate was the slow hydrolysis of the NHS-carbamate functionality, resulting in the formation of free NHS **C** and carbamic acid **D**.

Procedure: 1 mg of **6** was dissolved in 0.7 mL of fresh DMSO-d_6. Next, ^{1}H NMR spectra were recorded every 30 minutes for overall 6 h.

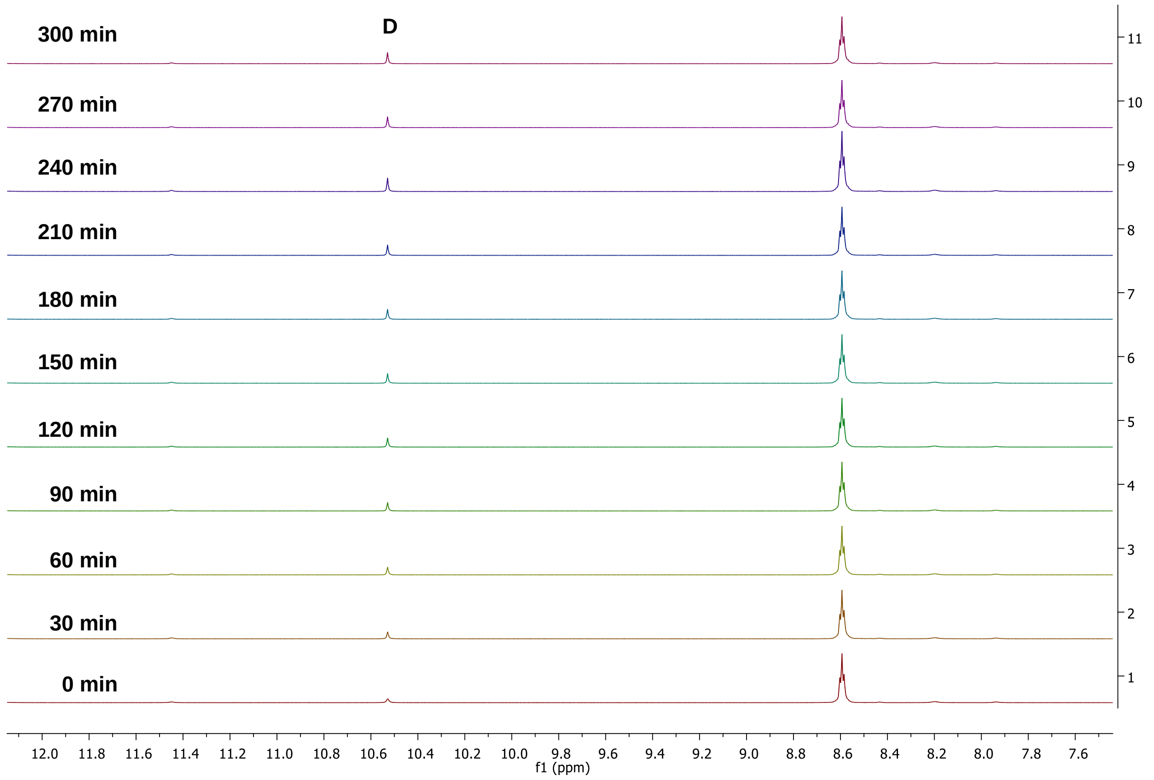
D
300 min
270 min
240 min
210 min
180 min
150 min
120 min
90 min
60 min
30 min
0 min
f1 (ppm)

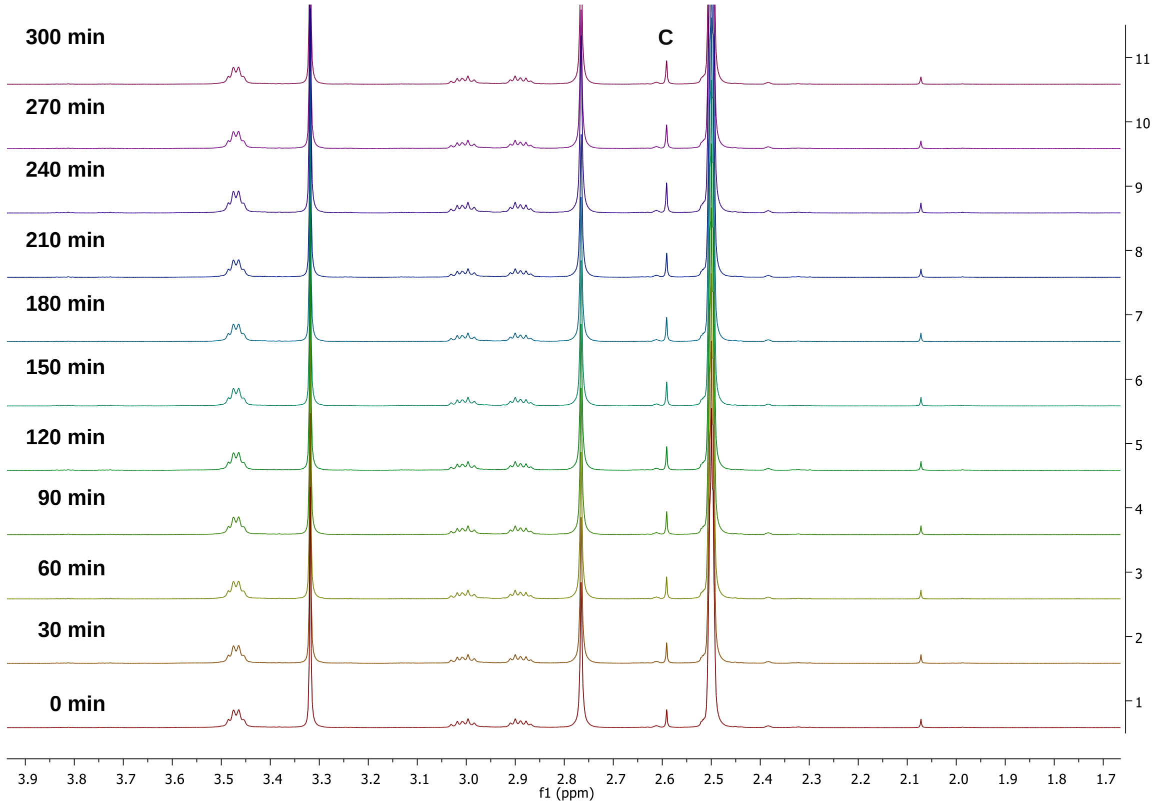
C
300 min
270 min
240 min
210 min
180 min
150 min
120 min
90 min
60 min
30 min
0 min
f1 (ppm)

Statistical Validation of Monolink Performance for DSSO vs DSSO-carbamate with 20S Proteasome Complex

DSSO-carbamate was found to recover more monolinks over three experimental replicates.

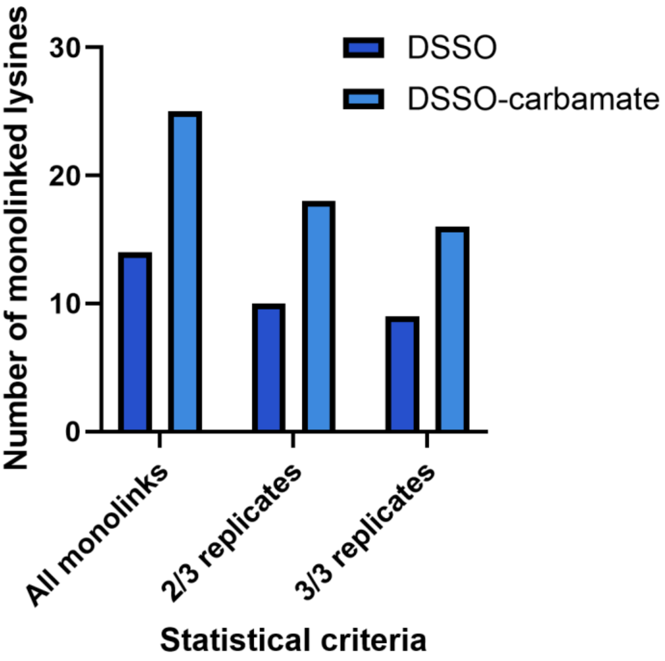

References

[1] Chowdhury, S.; Happonen, L.; Khakzad, H.; Malmström, L.; Malmström, Electron Cryo-Microscopy and Structural Modeling Approaches in Bacteria–Human Protein Interactions. *Med. Microbiol. Immunol. (Berl.)* **2020**, *209* (3), 265–275, doi.org/10.1007/s00430-020-00663-5. b). Chavez, J. D., Bruce J. E., Chemical cross-linking with mass spectrometry: a tool for systems structural biology, *Curr Opin Chem Biol.* **2019,** *48*, 8-18, doi.org/10.1016/j.cbpa.2018.08.006.

[2] Orbán-Németh, Z.; Beveridge, R.; Hollenstein, D. M.; Rampler, E.; Stranzl, T.; Hudecz, O.; Doblmann, J.; Schlögelhofer, P.; Mechtler, K. Structural Prediction of Protein Models Using Distance Restraints Derived from Cross-Linking Mass Spectrometry Data. *Nat. Protoc.* **2018**, *13* (3), 478–494, https://doi.org/10.1038/nprot.2017.146

[3] Yu, C.; Huang, L. Cross-Linking Mass Spectrometry (XL-MS): An Emerging Technology for Interactomics and Structural Biology. *Anal. Chem.* **2018**, *90* (1), 144–165, doi.org/10.1021/acs.analchem.7b04431.

[4] Piersimoni, L.; Kastritis, P. L.; Arlt, C.; Sinz, A. Cross-Linking Mass Spectrometry for Investigating Protein Conformations and Protein–Protein Interactions─A Method for All Seasons. *Chem. Rev.* **2022**, *122* (8), 7500–7531, doi.org/10.1021/acs.chemrev.1c00786.

[5] Matzinger, M.; Mechtler, K. Cleavable Cross-Linkers and Mass Spectrometry for the Ultimate Task of Profiling Protein–Protein Interaction Networks in Vivo. *J. Proteome Res.* **2021**, *20* (1), 78–93, doi.org/10.1021/acs.jproteome.0c00583. b). Piersimoni, L.; Sinz, A. Cross-Linking/Mass Spectrometry at the Crossroads. *Anal. Bioanal. Chem.* **2020**, *412* (24), 5981–5987, doi.org/10.1007/s00216-020-02700-x.

[6] Lui, F., Heck, A. J. R. Interrogating the architecture of protein assemblies and protein interaction networks by cross-linking mass spectrometry, *Curr. Opin. Struct. Biol.*, **2015**, *35*, 100-108, doi.org/10.1016/j.sbi.2015.10.006.

[7] Liko, I.; Allison, T. M.; Hopper, J. T.; Robinson, C. V. Mass Spectrometry Guided Structural Biology. *Curr. Opin. Struct. Biol.* **2016**, *40*, 136–144, doi.org/10.1016/j.sbi.2016.09.008.

[8] O'Reilly, F. J.; Rappsilber, J. Cross-Linking Mass Spectrometry: Methods and Applications in Structural, Molecular and Systems Biology. *Nat. Struct. Mol. Biol.* **2018**, *25* (11), 1000–1008. doi.org/10.1038/s41594-018-0147-0.

[9] Kao, A.; Chiu, C.; Vellucci, D.; Yang, Y.; Patel, V. R.; Guan, S.; Randall, A.; Baldi, P.; Rychnovsky, S. D.; Huang, L. Development of a Novel Cross-Linking Strategy for Fast and Accurate Identification of Cross-Linked Peptides of Protein Complexes *. *Mol. Cell. Proteomics* **2011**, *10* (1), doi.org/10.1074/mcp.M110.002212.

[10] Müller, M. Q.; Dreiocker, F.; Ihling, C. H.; Schäfer, M.; Sinz, A. Cleavable Cross-Linker for Protein Structure Analysis: Reliable Identification of Cross-Linking Products by Tandem MS. *Anal. Chem.* **2010**, *82* (16), 6958–6968. doi.org/10.1021/ac101241t.

[11] M. Burke, A.; Kandur, W.; J. Novitsky, E.; M. Kaake, R.; Yu, C.; Kao, A.; Vellucci, D.; Huang, L.; D. Rychnovsky, S. Synthesis of Two New Enrichable and MS-Cleavable Cross-Linkers to Define Protein–Protein Interactions by Mass Spectrometry. *Org. Biomol. Chem.* **2015**, *13* (17), 5030–5037. doi.org/10.1039/C5OB00488H.

[12] Nury, C.; Redeker, V.; Dautrey, S.; Romieu, A.; van der Rest, G.; Renard, P.-Y.; Melki, R.; Chamot-Rooke, J. A Novel Bio-Orthogonal Cross-Linker for Improved Protein/Protein Interaction Analysis. *Anal. Chem.* **2015**, *87* (3), 1853–1860. doi.org/10.1021/jasms.9b00085.
[13] Yu, C.; Kandur, W.; Kao, A.; Rychnovsky, S.; Huang, L. Developing New Isotope-Coded Mass Spectrometry-Cleavable Cross-Linkers for Elucidating Protein Structures. *Anal. Chem.* **2014**, *86* (4), 2099–2106, doi.org/10.1021/ac403636b.
[14] Steigenberger, B.; Albanese, P.; Heck, A. J. R.; Scheltema, R. A. To Cleave or Not To Cleave in XL-MS? *J. Am. Soc. Mass Spectrom.* **2020**, *31* (2), 196–206, doi.org/10.1021/jasms.9b00085.
[15] Sinnott, M.; Malhotra, S.; Madhusudhan, M. S.; Thalassinos, K.; Topf, M. Combining Information from Crosslinks and Monolinks in the Modeling of Protein Structures. *Structure* **2020**, *28* (9), 1061-1070.e3, doi.org/10.1016/j.str.2020.05.012.
[16] Bullock, J. M. A.; Thalassinos, K.; Topf, M. Jwalk and MNXL Web Server: Model Validation Using Restraints from Crosslinking Mass Spectrometry. *Bioinformatics* **2018**, *34* (20), 3584–3585, doi.org/10.1093/bioinformatics/bty366.
[17] Herzog, F.; Kahraman, A.; Boehringer, D.; Mak, R.; Bracher, A.; Walzthoeni, T.; Leitner, A.; Beck, M.; Hartl, F.-U.; Ban, N.; Malmström, L.; Aebersold, R. Structural Probing of a Protein Phosphatase 2A Network by Chemical Cross-Linking and Mass Spectrometry. *Science* **2012**, *337* (6100), 1348–1352, doi.org/10.1126/science.1221483.
[18] Chen, X.; Sailer, C.; Kammer, K. M.; Fürsch, J.; Eisele, M. R.; Sakata, E.; Pellarin, R.; Stengel, F. Mono- and Intralink Filter (Mi-Filter) To Reduce False Identifications in Cross-Linking Mass Spectrometry Data. *Anal. Chem.* **2022**, *94* [51), 17751–17756, doi.org/10.1021/acs.analchem.2c00494
[19] Manalastas-Cantos, K.; Adoni, K. R.; Pfeifer, M.; Märtens, B.; Grünewald, K.; Thalassinos, K.; Topf, M. Modeling Flexible Protein Structure with AlphaFold2 and Cross-Linking Mass Spectrometry. *Mol. Cell. Proteomics* **2024**, *0* [0), doi.org/10.1016/j.mcpro.2024.100724.
[20] Ser, Z.; Cifani, P.; Kentsis, A. Optimized Cross-Linking Mass Spectrometry for in Situ Interaction Proteomics. *J. Proteome Res.* **2019**, *18* (6), 2545–2558, doi.org/10.1016/j.mcpro.2024.100724.
[21] Fux, A.; Korotkov, V. S.; Schneider, M.; Antes, I.; Sieber, S. A. Chemical Cross-Linking Enables Drafting ClpXP Proximity Maps and Taking Snapshots of In Situ Interaction Networks. *Cell Chem. Biol.* **2019**, *26* (1), 48-59.e7, doi.org/10.1016/j.chembiol.2018.10.007.
[22] Liu, F.; Lössl, P.; Rabbitts, B. M.; Balaban, R. S.; Heck, A. J. R. The Interactome of Intact Mitochondria by Cross-Linking Mass Spectrometry Provides Evidence for Coexisting Respiratory Supercomplexes. *Mol. Cell. Proteomics MCP* **2018**, *17* (2), 216–232, doi.org/10.1074/mcp.RA117.000470.
[23] Fasci, D.; van Ingen, H.; Scheltema, R. A.; Heck, A. J. R. Histone Interaction Landscapes Visualized by Crosslinking Mass Spectrometry in Intact Cell Nuclei. *Mol. Cell. Proteomics MCP* **2018**, *17* (10), 2018–2033, doi.org/10.1074/mcp.RA118.000924.
[24] Bartolec, T. K.; Smith, D.-L.; Pang, C. N. I.; Xu, Y. D.; Hamey, J. J.; Wilkins, M. R. Cross-Linking Mass Spectrometry Analysis of the Yeast Nucleus Reveals Extensive Protein–Protein Interactions Not Detected by Systematic Two-Hybrid or Affinity Purification-Mass Spectrometry. *Anal. Chem.* **2020**, *92* (2), 1874–1882, doi.org/10.1021/acs.analchem.9b03975.
[25] Kaiser, D.; Klose, I.; Oost, R.; Neuhaus, J.; Maulide, N. Bond-Forming and -Breaking Reactions at Sulfur(IV): Sulfoxides, Sulfonium Salts, Sulfur Ylides, and Sulfinate Salts. *Chem. Rev.* **2019**, *119* (14), 8701–8780, doi.org/10.1021/acs.chemrev.9b00111.
[26] Zhang, L.; Wu, S.; Feng, Y.; Wang, D.; Jia, X.; Liu, Z.; Liu, J.; Wang, W. Ligand-Bound Glutamine Binding Protein Assumes Multiple Metastable Binding Sites with Different Binding Affinities. *Commun. Biol.* **2020**, *3* (1), 1–11, doi.org/10.1038/s42003-020-01149-z.
[27] Meng, E. C.; Goddard, T. D.; Pettersen, E. F.; Couch, G. S.; Pearson, Z. J.; Morris, J. H.; Ferrin, T. E. UCSF ChimeraX: Tools for Structure Building and Analysis. *Protein Sci. Publ. Protein Soc.* **2023**, *32* (11), e4792, doi.org/10.1002/pro.4792.
[28] Fulmer, G. R.; Miller, A. J. M.; Sherden, N. H.; Gottlieb, H. E.; Nudelman, A.; Stoltz, B. M.; Bercaw, J. E.; Goldberg, K. I. NMR Chemical Shifts of Trace Impurities: Common Laboratory Solvents, Organics, and Gases in Deuterated Solvents Relevant to the Organometallic Chemist. *Organometallics* **2010**, *29* (9), 2176–2179, doi.org/10.1021/om100106e.
